# Interventions to Facilitate Shared Decision-Making Using Decision Aids with Coronary Heart Disease Patients: Systematic Review and Meta-Analysis

**DOI:** 10.31083/j.rcm2408246

**Published:** 2023-08-25

**Authors:** Haoyang Zheng, Duo Zhang, Wei Xiang, Yuxi Wu, Zesheng Peng, Yong Gan, Shengcai Chen

**Affiliations:** ^1^Department of Neurosurgery, Union Hospital, Tongji Medical College, Huazhong University of Science and Technology, 430022 Wuhan, Hubei, China; ^2^Department of Nursing, Tongji Hospital, Tongji Medical College, Huazhong University of Science and Technology, 430030 Wuhan, Hubei, China; ^3^Department of Social Medicine and Health Management, School of Public Health, Tongji Medical College, Huazhong University of Science and Technology, 430030 Wuhan, Hubei, China; ^4^Department of Neurology, Union Hospital, Tongji Medical College, Huazhong University of Science and Technology, 430022 Wuhan, Hubei, China

**Keywords:** shared decision making, patient decision aids, coronary heart disease, systematic review, meta-analysis

## Abstract

**Background:**

Coronary heart disease (CHD) is the leading cause of death in the world. There are some decision-making conflicts in the management of chest pain, treatment methods, stent selection, and other aspects due to the unstable condition of CHD in the treatment stage. Although using decision aids to facilitate shared decision-making (SDM) contributes to high-quality decision-making, it has not been evaluated in the field of CHD. This review systematically assessed the effects of SDM in patients with CHD.

**Methods:**

We conducted a systematic review and meta-analysis of randomized controlled trials of SDM interventions in patients with CHD from database inception to 1 June 2022 (PROSPERO [Unique identifier: CRD42022338938]). We searched for relevant studies in the PubMed, Embase, Cochrane Library, Web of Science, CNKI, and Wan Fang databases. The primary outcomes were knowledge and decision conflict. The secondary outcomes were satisfaction, patient participation, trust, acceptance, quality of life, and psychological condition.

**Results:**

A total of 8244 studies were retrieved. After screening, ten studies were included in the analysis. Compared with the control group, SDM intervention with patient decision aids obviously improved patients’ knowledge, decision satisfaction, participation, and medical outcomes and reduced decision-making conflict. There was no significant effect of SDM on trust.

**Conclusions:**

This study showed that SDM intervention in the form of decision aids was beneficial to decision-making quality and treatment outcomes among patients with CHD. The results of SDM interventions need to be evaluated in different environments.

## 1. Introduction

Cardiovascular disease (CVD) is the leading cause of death globally, accounting for 16% of mortality globally [1]. Coronary heart disease (CHD) is one of the main CVDs. Its primary clinical manifestation is retrosternal pain, and its main etiology is lifestyle changes and psychological factors [2]. The incidence and mortality of CHD are increasing yearly in developing countries [3]. In China, the number of patients with CHD was about 11.39 million, with a mortality rate as high as 248.42/100,000 [4], and it is estimated that the prevalence rate will reach 1895.91/100,000 in 2025 [5].

At present, the main treatment methods for CHD include optimal medical therapy (OMT), percutaneous coronary intervention (PCI), and coronary artery bypass grafting (CABG) [6]. The complexity of the disease and individual differences lead to the comparability of treatment methods for CHD. For example, compared with PCI, OMT cannot reduce long-term mortality and the risk of cardiovascular events in most patients with stable CHD [7]. Compared with bare metal stents (BMS), drug-eluting stents (DES) cannot improve survival or long-term quality of life of patients [8]. CABG can effectively enhance the quality of life of severe CHD patients, but the survival advantage in stable CHD patients is uncertain [9]. It is important to note that the risks and benefits of different treatments vary depending on the patient’s age, complications, and degree of vascular stenosis. For example, CABG’s risks and benefits differ for older and younger adults [10]. CHD treatment decision-making is a “sensitive preference” choice, and the best decision depends on the decision-making scenario of patients’ response to the outcome probability [11]. Studies have shown that patients with stable CHD have apparent decision-making conflict, and the effective rate of decision-making accounts for 51.32% [12]. Patients’ choice of stent type was consistent with the actual implantation type, accounting for only 50% [13]. A survey of patients undergoing PCI in China showed that only 28.0% of patients implanted with PCI were in line with medical practice [14]. Doctors’ preferences and clinical experience likely affected the final decision-making results. It is worth considering whether patients are aware of alternative options and relevant information and whether they have the opportunity to express their preferences and expectations to doctors [15]. The treatment of CHD should consider not only the potential survival advantages but also the possibility of symptom relief, the anticipation of quality of life, and the preferences of patients. Compared with a lack of tailored treatment options, informing the risks and benefits according to the characteristics of patients can help them understand the disease information and make the most appropriate choice.

Shared decision-making (SDM) is the key to improving the quality of decision-making and extending the concept of “patient-centered”. SDM is a process in which doctors, nurses, and patients can fully share and understand disease information. Patients can provide timely feedback values and jointly determine the medical results after discussion [16, 17]. Patient decision aids (PtDAs) can help doctors and patients achieve win-win SDM. It is an evidence-based tool to provide information for patients [18]. It is suitable for “sensitive preference” decision-making scenarios [19]. There are many forms of decision-making aids, mainly including booklets, videos, applications, *etc*. Such as Ottawa Decision Support Framework (https://decisionaid.ohri.ca/), Mayo Clinic Shared Decision-Making National Resource Center (https://carethatfits.org/shared-decision-making/), *etc*. Using PtDAs to promote SDM can help patients better participate in the decision-making process, improve the knowledge level of disease, and reduce decision-making conflicts [20]. As a specialty discipline, cardiology focuses on creating and using state-of-the-art evidence-based PtDAs to promote SDM [21]. SDM in the cardiovascular field mainly focuses on clinical scenarios such as atrial fibrillation (AF), CHD, and heart failure (HF) [22]. These studies mostly use manuals or videos for decision-making assistance, requiring patients to have sufficient decision-making ability and time. While these studies have played a driving role in the development of SDM in cardiology, they still face issues such as uncertain timing of the use of SDM, time and resource constraints, and how doctors understand patient preferences.

Optimizing treatment decisions for CHD is crucial. In 2019, the American College of Cardiology and American Heart Association Guideline on the Primary Prevention of Cardiovascular Disease emphasized the co-guidance of clinical judgment and patient preference in CHD [23]. The published literature reports on the application of SDM in CHD from aspects such as influencing factors, development of PtDAs, and application effectiveness [24]. However, there are significant differences in patients’ willingness to participate, low enthusiasm among doctors, poor research results, and even contradictions [15]. There was controversy in some studies regarding the effectiveness of shared decision-making in coronary heart disease, such as decision conflicts, knowledge, and decision satisfaction [15, 23]. Thus, it is necessary to integrate these studies and comprehensively evaluate the intervention effect of SDM in CHD. We conducted a systematic review and meta-analysis of randomized controlled trials (RCTs) to assess whether SDM in the form of PtDAs can improve the quality of decision-making and treatment outcomes in patients with CHD to provide a reference for the development of SDM in clinical cardiology practice.

## 2. Materials and Methods

### 2.1 Design

We conducted this systematic review and meta-analysis following the Preferred Reporting Items for Systematic Reviews and Meta-Analyses guidelines (**Supplementary Material 1**) and registered at PROSPERO (CRD42022338938). This review retrieved and synthesized public data from published studies and did not require ethical approval or review.

### 2.2 Search Strategy

A comprehensive literature search was conducted using the PubMed, Embase, Cochrane Library, Web of Science, CNKI, and Wan Fang databases from their inception to 1 June 2022. We used search strategies corresponding to each database to maximize sensitivity and also reviewed the relevant articles’ references to identify other potentially eligible articles. This search algorithm was shown in **Supplementary Material 2**.

### 2.3 Eligibility and Exclusion Criteria

In this review, we included RCTs of SDM intervention for CHD. The inclusion criteria were defined according to the population, intervention, comparison, and outcome in the following format:

P (Population): The study population included patients requiring diagnosis and treatment of CHD, including patients requiring diagnosis of CHD due to chest pain or other symptoms suggestive of CHD. Patients who have been diagnosed with CHD and need to make treatment decisions.

I (Intervention): This study defines SDM using PtDAs as an intervention that helps CHD patients participate in SDM regarding diagnosis and treatment. The PtDAs had no limitation in form.

C (Comparison): The control group received usual care or other forms of health education.

O (Outcome): The primary outcomes included knowledge, and decision conflict [25] (a state of uncertainty about the course of action to take, which likely occurs when the choices involve risk or uncertainty of outcomes, high risk in potential gains and losses, the need to make value trade-offs, and the regret over the positive aspects of rejected options). The secondary outcomes included satisfaction (patients’ satisfaction with the format, content, and decision-making process of the PtDAs), patient participation, acceptability of using the PtDAs, and trust in physicians and others.

We excluded letters, case reports, comments, reviews, and conference abstracts.

### 2.4 Study Selection

After removing duplicates, two reviewers independently applied eligibility and exclusion criteria to screen the titles and abstracts of each article for initial eligibility and to screen the full texts for final eligibility. All disagreements were solved through consensus adjudication, with final confirmation of exclusion or inclusion by the third reviewer.

### 2.5 Data Extraction

The research team used a specialized datasheet for data extraction. Two researchers independently conducted data extraction. Extraction contents included the following aspects: (a) Basic information: the first author, publication year, and country. (b) Research contents: study design, decision-making problems, inclusion and exclusion criteria, and sample size. (c) Intervention measures: characteristics of the PtDAs, intervention measures, and control measures; and (d) Outcome indicators: the primary outcomes were knowledge and decision conflict; the secondary indicators were satisfaction, patient participation, acceptability of the PtDAs, trust in physicians and others.

### 2.6 Quality Assessment

Two reviewers independently assessed the possible risk of bias for each study, at the outcome and study level, using the Cochrane Collaboration revised tool to determine the risk of bias in randomized trials (RoB 2) [26]. We assessed the quality of individual studies, following the tools structured in 5 domains: bias arising from the randomization process; due to deviations from intended interventions; missing outcome data; measurement of the outcome; and selection of the reported result. Each domain was rated as having a low risk of bias, some concerns, or a high risk of bias.

### 2.7 Data Analysis

Mean differences (MDs) were used for continuous variables measured using the same instrument and scales, and standardized mean differences (SMDs) were used for similar outcomes assessed by different instruments. The heterogeneity test was assessed by $I^{2}$ test. All results showed a high heterogeneity ($I^{2}$ > 50); therefore, the random-effect models were used for all analyses. Visual funnel plots and the Egger’s test assessed publication bias of the primary outcome indicators. We conducted a subgroup analysis based on different countries to account for potential differences across countries. To identify the potential sources of heterogeneity, we also conducted a sensitivity analysis to determine the stability of the results by omitting individual data one by one (leave-one-out analysis) to ensure that the conclusions were stable and reliable.

## 3. Results

### 3.1 Search Results

**Fig. 1.**
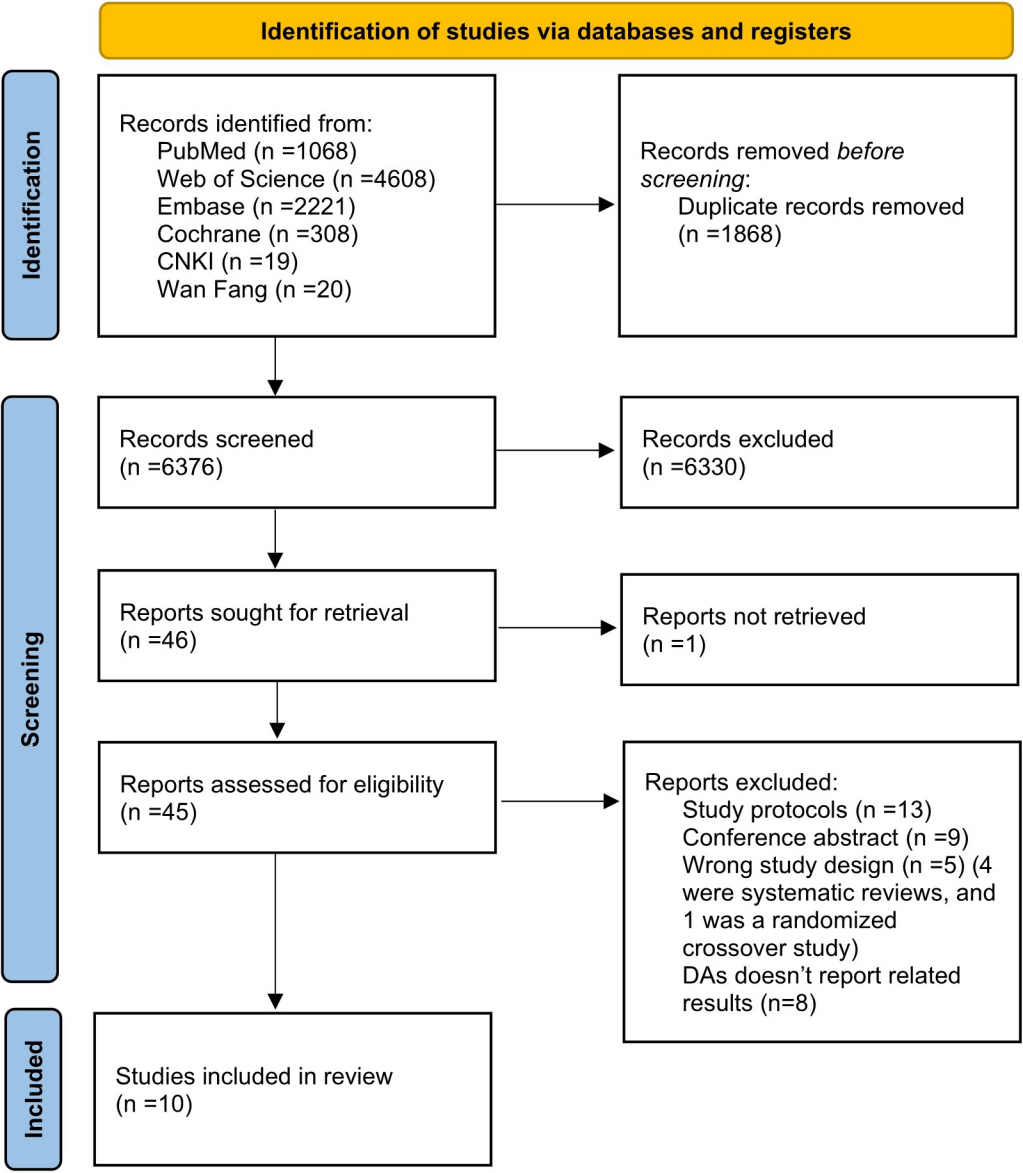
**Flow diagram: PRISMA flow diagram of the literature search, identification, screening, and inclusion in the systematic review and meta-analysis**. DAs, decision aids.

The study selection process and results are shown in Fig. 1. We identified 8244 reports from six databases. Other pathways found no additional articles. After removing 1868 duplicates, we screened the titles and abstracts of 6376 studies according to prespecified inclusion and exclusion criteria. After screening the full text of 45 studies, ten were included in this review [27, 28, 29, 30, 31, 32, 33, 34, 35, 36].

### 3.2 Study Characteristics

The characteristics of the included studies are summarized in Table 1 (Ref. [27, 28, 29, 30, 31, 32, 33, 34, 35, 36]). All the included studies were published between 2000 and 2022. Five studies were conducted in America [28, 29, 30, 31, 32], one in Canada [27], and four in China [33, 34, 35, 36]. There were 2133 participants, with sample sizes ranging from 90 to 898.

**Table 1.** **General characteristics of included RCTs**.

Study, year	Country	Inclusion and exclusion criteria	No. of patients randomized	Control arm			Intervention arm			Outcomes
				Control group	No. of patients	Mean age, y	Intervention group	No. of patients	Mean age, y	
Morgan *et al*. [27] (2000)	Canada	Inclusion criteria: patients were diagnosed with ischemic heart disease by angiography, which was defined as more than 50% narrowing of at least one coronary artery and could be treated by elective revascularization (bypass surgery and/or angioplasty) with the option of ongoing medical therapy.	187	Patients did not receive any additional educational material from the study investigators.	97	60	Using decision aids (DAs) to enhance patients’ knowledge of the benefits and harms of different treatment alternatives.	90	60	Satisfaction, knowledge, treatment preference
Hess *et al*. [28] (2012)	USA	Eligible patients included adults aged older than 17 years who presented to the emergency department (ED) with primary symptoms of nontraumatic chest pain and who were being considered for admission to the ED for monitoring and cardiac stress testing within 24 hours.	204	Clinicians will discuss the results of the diagnostic investigations and management options with the patient in usual fashion. No research-related interventions will be administered.	103	54.9	DAs enable the patient to decide whether to perform an urgent cardiac stress test or follow up with the physician within 72 hours.	101	54.5	Knowledge, decisional conflict, degree of trust in the physician, acceptability, patient engagement, satisfaction, a major adverse cardiac event, decision behavior
Coylewright *et al*. [29] (2016)	USA	Patients were adults (aged ≥18 years) who were considered candidates for both optimal medical therapy and percutaneous coronary intervention (PCI) for the treatment of stable coronary artery disease by the referring clinician.	124	Usual care	59	67.9	DAs were stratified by angina type (CCS I-II angina *vs*. III angina) for use by patients.	65	68.5	Knowledge, decisional conflict, patient engagement
Hess *et al*. [30] (2016)	USA	Eligible patients included adults (aged >17 years) presenting to the emergency department with a chief complaint of chest pain who were being considered for cardiac stress testing or coronary computed tomography angiography.	898	No research-related interventions will be administered.	447	50.6	DAs to enable patients to participate in the decision to undergo urgent cardiac stress testing or follow-up with a physician in 24-72 hours.	451	50.0	Knowledge, decisional conflict, degree of trust in the physician, patient engagement, satisfaction, acceptability, clinical outcome, decision behavior
Case* et al*. [31] (2019)	USA	Patients older than 18 years of age who presented to the hospital with chest pain or other potential coronary heart disease (CHD) symptoms and no known history of CHD or previous assessment for CHD.	90	Received standard of care.	49	53.1	Patients were provided an iPad with the DAs website before their office visit.	50	49.9	Knowledge, decisional conflict, degree of trust in the physician, patient engagement, acceptability
Doll *et al*. [32] (2019)	USA	Subjects were adult patients with chronic stable angina, chest pain or angina with a positive functional test, unstable angina, or non-ST-segment elevation myocardial infarction.	203	Usual care	100	63.9	Patients used the web-based decision aid or printed copies.	103	63.3	Knowledge, decision-making preference, treatment preference, decisional conflict
Wu *et al*. [33] (2020)	China	Patients diagnosed with CHD, aged ≥18 years, could participate in shared decision-making (SDM) without severe impairment of vision, hearing, and comprehension.	124	Patients were given usual care, including a comprehensive admission evaluation and medication or PCI.	62	53.9	DAs helped patients prioritize alternative treatment options.	62	54.3	Decisional conflict, patient engagement, satisfaction
Li *et al*. [34] (2021)	China	Patients had a Hamilton Anxiety Scale score >14, met the diagnostic criteria for CHD, and were older than 60 years.	123	Patients received routine disease education.	60	66.7	In addition to conventional education, DAs helped patients Shared decisions through video and graphic materials.	63	67.4	Hope Index, satisfaction, specific medication beliefs
Ren *et al*. [35] (2022)	China	Patients diagnosed with CHD, aged ≥60 years, could participate in SDM without severe impairment of vision, hearing, and comprehension.	90	Patients received knowledge and psychological counseling about CHD.	45	66.8	Patients received health manuals, oral education, videos, lectures and other diversified forms.	45	67.2	Knowledge, health behavior, sleep quality, disease control effectiveness
Wu *et al*. [36] (2022)	China	The patients (aged ≥60 years) met the diagnostic criteria for stable CHD and the New York Heart Association cardiac function classification II to IV.	90	Patients were given usual care, including a comprehensive admission evaluation.	45	72.4	Patients received psychological suggestion and share decision-making options.	45	72.2	Health behavior capacity, resilience, decisional conflict, self-efficacy, clinical outcome

Note: y, years; CCS, Canadian Cardiovascular Society; CHD, coronary heart disease; DAs, decision aids; ED, emergency department; PCI, percutaneous coronary intervention; RCTs, randomized controlled trials; SDM, shared decision-making.

### 3.3 The Format and Content of PtDAs

PtDAs varied considerably in their basic theoretical framework, form, and methods of expression. We identified three formats of PtDAs, as shown in Table 2 (Ref. [27, 28, 29, 30, 31, 32, 33, 34, 35, 36]). Six studies used paper formats [28, 29, 30, 33, 35, 36], two used video programs [27, 34], and two were web-based [31, 32]. Except for one study [34], which did not explain how to carry out the PtDAs design conception and process, the other studies introduced the design process of PtDAs and analyzed information and treatment options in patients with CHD. The content of CHD in PtDAs varied with different research methods.

**Table 2.** **The format and content of PtDAs**.

Study, year	Format	Content	Development basis
Morgan *et al*. [27] (2000)	An interactive video program and a brochure	Presented information about the possible risks and benefits associated with three treatment alternatives for ischemic heart disease: medical therapy, bypass surgery, and angioplasty.	The Decision-Making Program was produced by the Foundation for Informed Medical Decision Making.
Hess *et al*. [28] (2012)	Booklet (text and diagrams)	(a) Describes for patients the rationale and results of the initial evaluation as well as the rationale for further cardiac stress testing, (b) depicts the patient’s pretest probability of acute coronary syndromes within 45 days using a risk communication pictograph.	The Ottawa Framework for Shared Decision Making and self-determination theory.
Coylewright *et al*. [29] (2016)	A paper-based decision aid	Describes the possible risks and benefits associated with optimal medical therapy and percutaneous coronary intervention (PCI).	Using a practice-based, patient-centered, and participatory approach to design PCI choice, requiring multidisciplinary input from clinicians, health service researchers, design experts, statisticians, and patients.
Hess *et al*. [30] (2016)	Booklet (text and diagrams) and The pretest consult instrument	Describes the rationale and results of the initial evaluation, identify personalized 45-day risk for acute coronary syndrome.	The Ottawa Framework for Shared Decision Making and self-determination theory.
Case *et al*. [31] (2019)	An interactive, web-based tool	Provides information about coronary heart disease (CHD), as well as various tests used in its clinical evaluation.	The decision aid (DA) was hosted on a website developed and maintained by Georgetown University and created by a multifaceted team involving a steering group.
Doll *et al*. [32] (2019)	A web-based application	Describe the information of CHD and treatment options, as well as the benefits and risks of medical therapy, PCI, and coronary artery bypass grafting.	The DA was designed and developed by Duke University Medical Center clinicians.
Wu *et al*. [33] (2020)	Booklet (SDM intervention table)	Introduce various treatment options and costs of CHD, and explain the risks and benefits of each.	This study referred to the “Patient-centered Decision Aid” designed and developed by Coylewright *et al*. [29].
Li *et al*. [34] (2021)	Video and graphic materials	Introduce disease knowledge, treatment options, treatment benefits and possible risks.	No explanation of how to proceed with DA design process.
Ren *et al*. [35] (2022)	Decision aid brochure	Introduce the etiology, pathogenesis, diagnosis and treatment of CHD.	Implement nursing intervention combined with shared decision intervention based on chronic disease trajectory model.
Wu *et al*. [36] (2022)	Booklet (decision plan table)	Introduce the various treatment options for CHD, and explain the risks and benefits of each.	An intervention team was set up, including 1 director of cardiology, 1 attending doctor, 1 head nurse and 4 nurses, to make the decision plan table together.

Note: CHD, coronary heart disease; DA, decision aid; PCI, percutaneous coronary intervention; PtDAs, patient decision aids; SDM, shared decision-making.

Eight studies [27, 29, 31, 32, 33, 34, 35, 36] involved OMT, PCI, and CABG to treat CHD patients, while two [28, 30] focused on assessing the risk of developing the disease during diagnosis. All included study subjects were aware and had some degree of decision-making ability. The main content included in PtDAs was summarized as follows: (1) introducing the etiology, pathogenesis, and preventive measures of CHD; (2) explaining the characteristics, advantages, and possible risks of different treatment options through videos, pictures, graphic interpretation, and other forms; (3) helping patients clarify their values and preferences. For example, some scales allow patients to evaluate the significance of various potential gains and losses and factors (such as treatment time and cost), and provide a quantitative summary of patient preferences to guide their value trade-offs and decisions; (4) giving psychological counseling and emotional support, the use of positive psychology nursing and other methods to help patients establish an optimistic attitude; (5) conducting comprehensive communication with patients and their families to reach consensus on decision-making.

### 3.4 Quality Assessment of the Included Studies

**Fig. 2.**
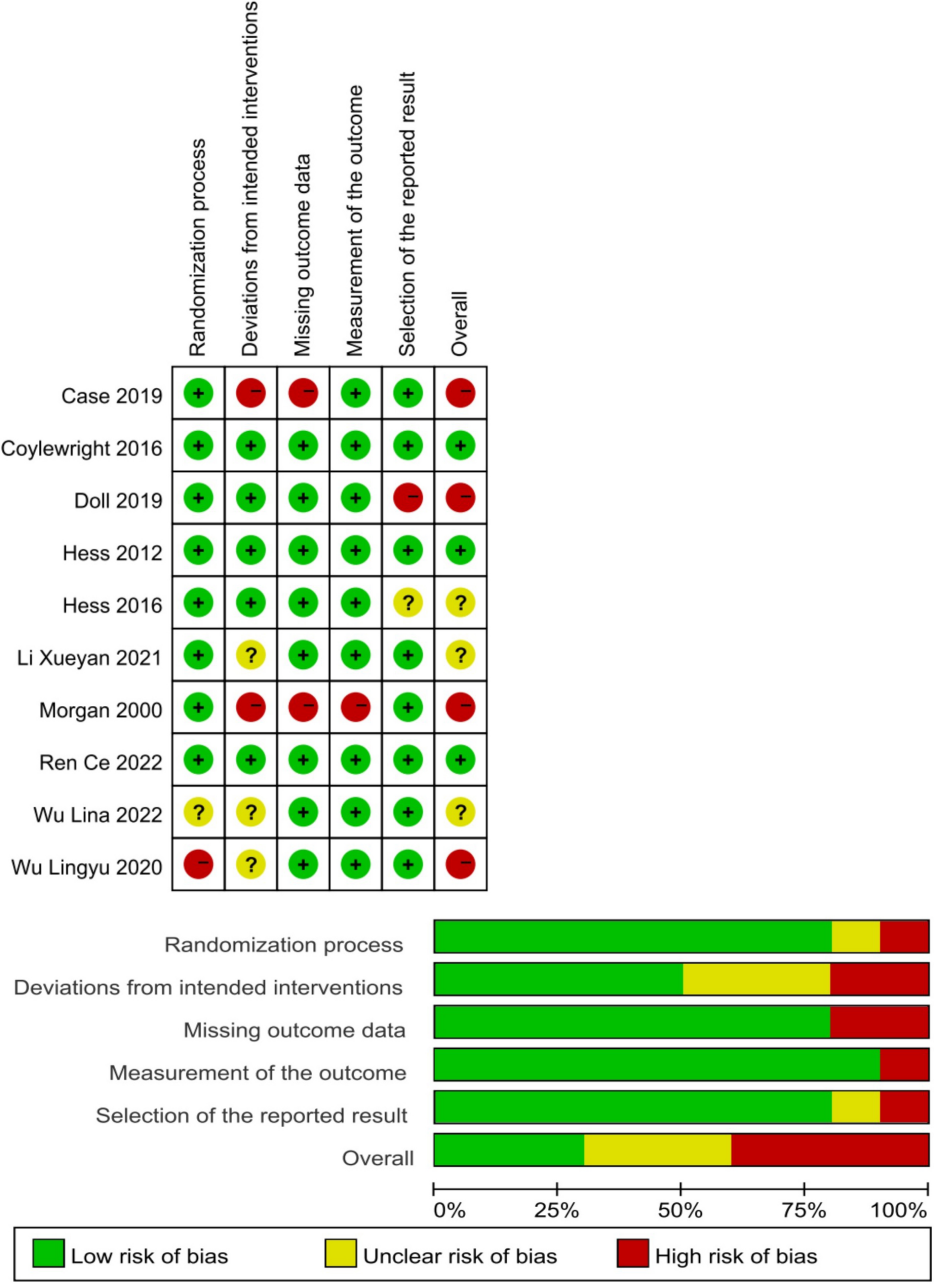
**Risk of bias: study quality was assessed according to the revised Cochrane risk of bias tool for randomized trials**.

We used the revised Cochrane risk of bias tool for randomized trials (RoB 2) to assess the risk of bias in each study, and the results are shown in Fig. 2. Details of randomization methods were reported in eight studies [27, 28, 29, 30, 31, 32, 34, 35], which resulted in a low risk of generating random sequences. Five of ten studies [28, 29, 30, 32, 35] reported blinding, which was considered to carry a low risk of bias. Due to the nature of the PtDAs, it must be acknowledged that it was not easy to deceive patients and crews adequately. Therefore, two studies [27, 31] that could deviate from the intended intervention were rated as having a high risk of bias; the other three studies [33, 34, 36] did not state specific measures of the study and were rated as some concerns. Nine studies [27, 28, 29, 31, 32, 33, 34, 35, 36] were considered to have a low risk of selective reporting bias because the programs were publicly registered, or the impact of any reporting bias could not be assessed. Another study [30] was some concern about selective reporting bias, and there were other potential sources of bias at low risk. One study [32] was a cluster-randomized trial in which a new module (identification/recruitment bias) was added in addition to the general modules to reduce the bias generated during recruitment. The new module was assessed as having a low risk of bias [32].

### 3.5 The Effect Size of SDM

#### 3.5.1 Primary Outcomes

3.5.1.1 Knowledge

**Fig. 3.**
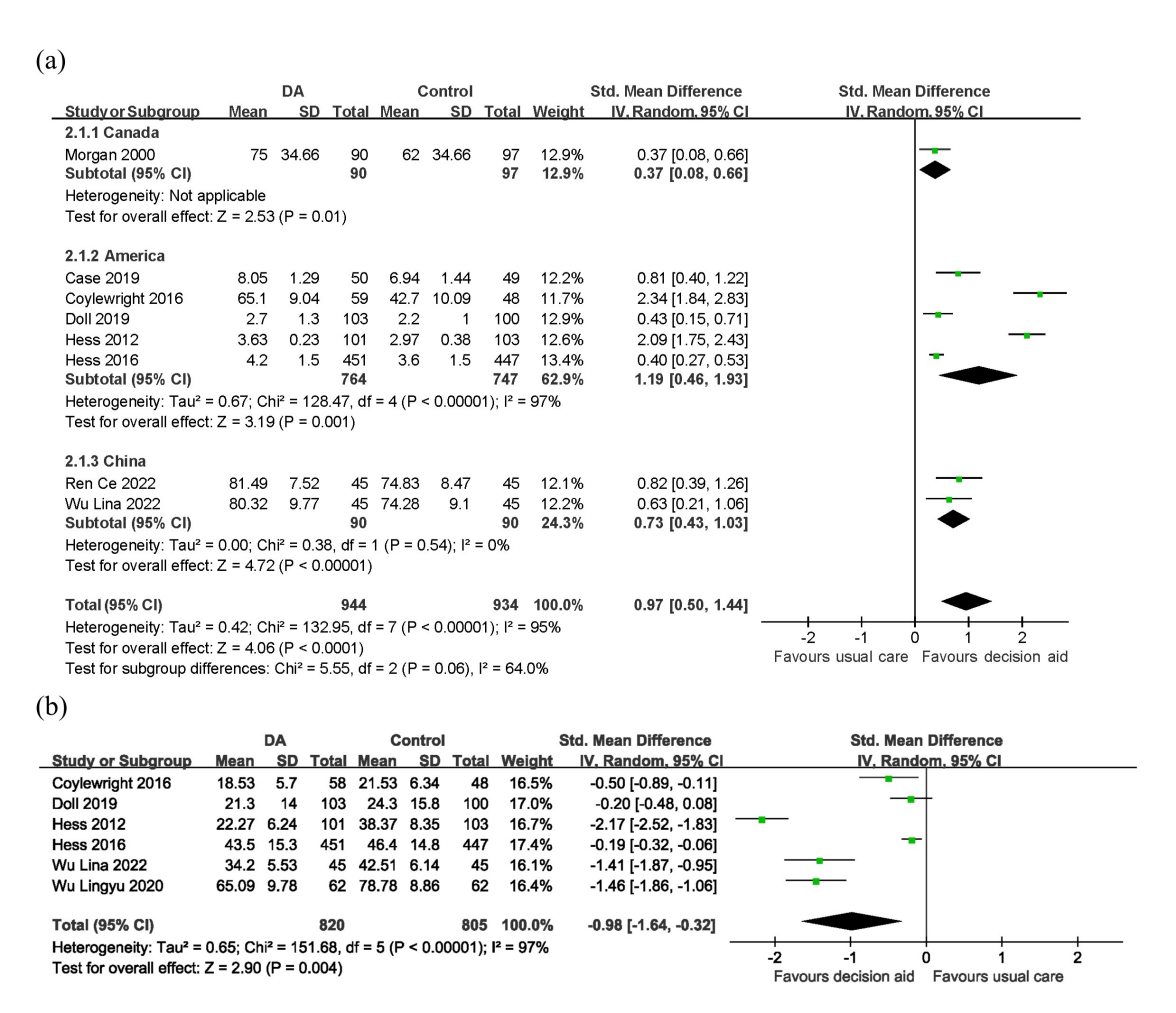
**Forest plots: (a) knowledge; (b) decision conflict**. DA, decision aid.

All studies mentioned that SDM improved patients’ knowledge, eight of which were included in the meta-analysis [27, 28, 29, 30, 31, 32, 35, 36]. A significant trend toward improved knowledge after applying the PtDAs intervention was found in the meta-analysis (SMD = 0.97; 95% CI: 0.50 to 1.44; $I^{2}$ = 95%; *p* < 0.0001; Fig. 3a). Another two studies were not included in the meta-analysis because they did not measure knowledge individually or use proprietary scales.

3.5.1.2 Decision Conflict

Seven studies [28, 29, 30, 31, 32, 33, 36] examined the impact of the SDM on decision conflict, six of which [28, 29, 30, 32, 33, 36] were included in the meta-analysis, and one [31] was excluded because it did not provide specific data. There was a significant difference in decision conflict (SMD = –0.98; 95% CI: –1.64 to –0.32; $I^{2}$ = 97%; Fig. 3b). This showed that SDM could effectively decrease the decision conflict (*p* = 0.004). Similarly, another study [31] that was not included in the meta-analysis reported that patients in the SDM group had a statistically significant reduction in decision conflict compared to the standard care group (*p* < 0.001).

#### 3.5.2 Secondary Outcomes

3.5.2.1 Satisfaction

**Fig. 4.**
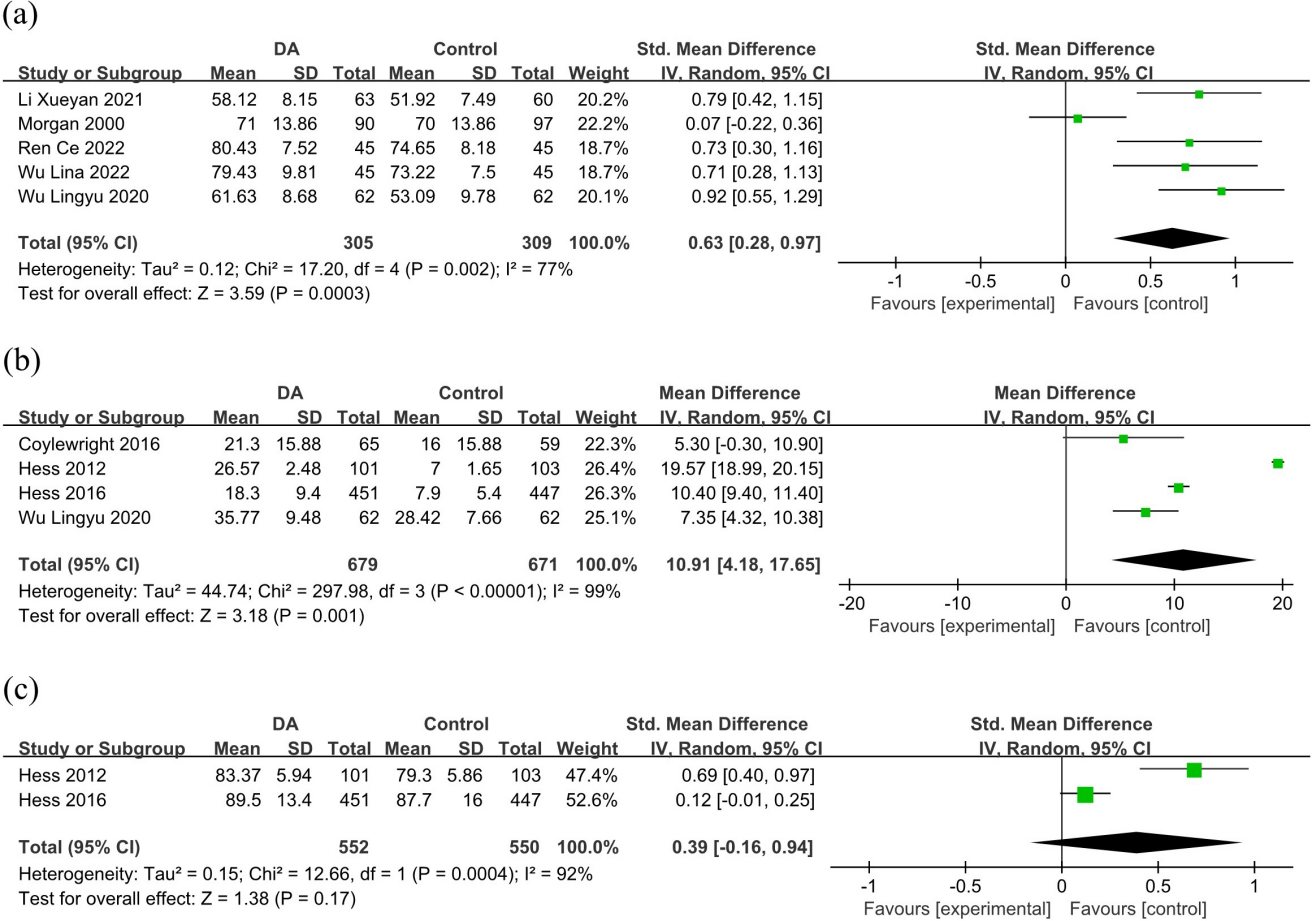
**Forest plots: (a) satisfaction; (b) patient participation; (c) trust**. DA, decision aid.

Eight studies [27, 28, 30, 31, 33, 34, 35, 36] reported patients’ satisfaction with the format and content of the SDM and decision-making process. Five studies [27, 33, 34, 35, 36] were included in the meta-analysis. This meta-analysis based on five studies showed that there was a significant difference in satisfaction (SMD = 0.63; 95% CI: 0.28 to 0.97; *p* = 0.0003; $I^{2}$ = 77%; Fig. 4a). Likewise, Hess *et al*. [28] reported that patients who used the SDM showed greater satisfaction with the decision-making process (strongly agree, 61% *vs*. 40%; 95% CI: 7% to 33%). However, although the results of another two studies [30, 31] showed that the experimental group reported high patient satisfaction (*p* = 0.06; *p* = 0.42), there was no statistical difference between the groups.

3.5.2.2 Patient Participation

Four studies [28, 29, 30, 33] were included in this meta-analysis to determine the degree of patient participation in the decision-making process. The meta-analysis showed a significant difference in patient participation (SMD = 10.91; 95% CI: 4.18 to 17.65; *p* = 0.001; $I^{2}$ = 99%; Fig. 4b). The patient participation in clinical decision-making was significantly greater in the SDM arm than in the usual care arm.

3.5.2.3 Trust

Three studies [28, 30, 31] assessed patients’ trust in their physicians after the intervention, with two studies [28, 30] included in the meta-analysis. The pooled SMD was 0.39 (95% CI: –0.16 to 0.94; Fig. 4c). This showed that the SDM did not effectively improve patients’ trust in their physicians (*p* = 0.17). Likewise, another study [31] showed that both the experimental and control groups reported high levels of patients’ trust in their physicians. There was no statistical difference between two groups (*p* = 0.26).

3.5.2.4 Other Outcomes

Three studies [28, 30, 31] revealed patients’ acceptance of implementing the SDM. Consistent results suggested that most participants thought the format and content of the SDM were appropriate, the information provided was straightforward to understand, and they were willing to recommend the SDM to others.

Adverse outcomes and living quality were evaluated by four studies [28, 30, 35, 36], and the results consistently showed that SDM could promote the treatment of disease and bring favorable outcomes to patients. Among these reports, one study [30] showed that patients who received the SDM were significantly less likely to decide to go to the emergency department observation unit for coronary computed tomography angiography or cardiac stress testing within 30 days. Likewise, two studies [35, 36] reported that after the intervention of the SDM, the scores of disease cognition, physical activity limitation, stable state of angina pectoris, and angina pectoris attack in the experimental group were higher than those in the control group (*p* < 0.05).

Two studies [34, 36] reported the psychological effects of SDM on patients. One study [34] showed that there were statistically significant differences in patients’ positive attitudes toward reality and the future, positive actions taken, intimate relationship scores with others, and total scores of the scale (*p* < 0.05). After the intervention, the scores of patients in the experimental group were higher than those before the intervention and control group. Another study [36] showed that the Connor-Davidson resilience scale (CD-RISC) score of patients in the SDM group was higher than that in the control group, with statistically significant differences (*p* < 0.05).

### 3.6 Publication Bias and Sensitivity Analysis

**Fig. 5.**
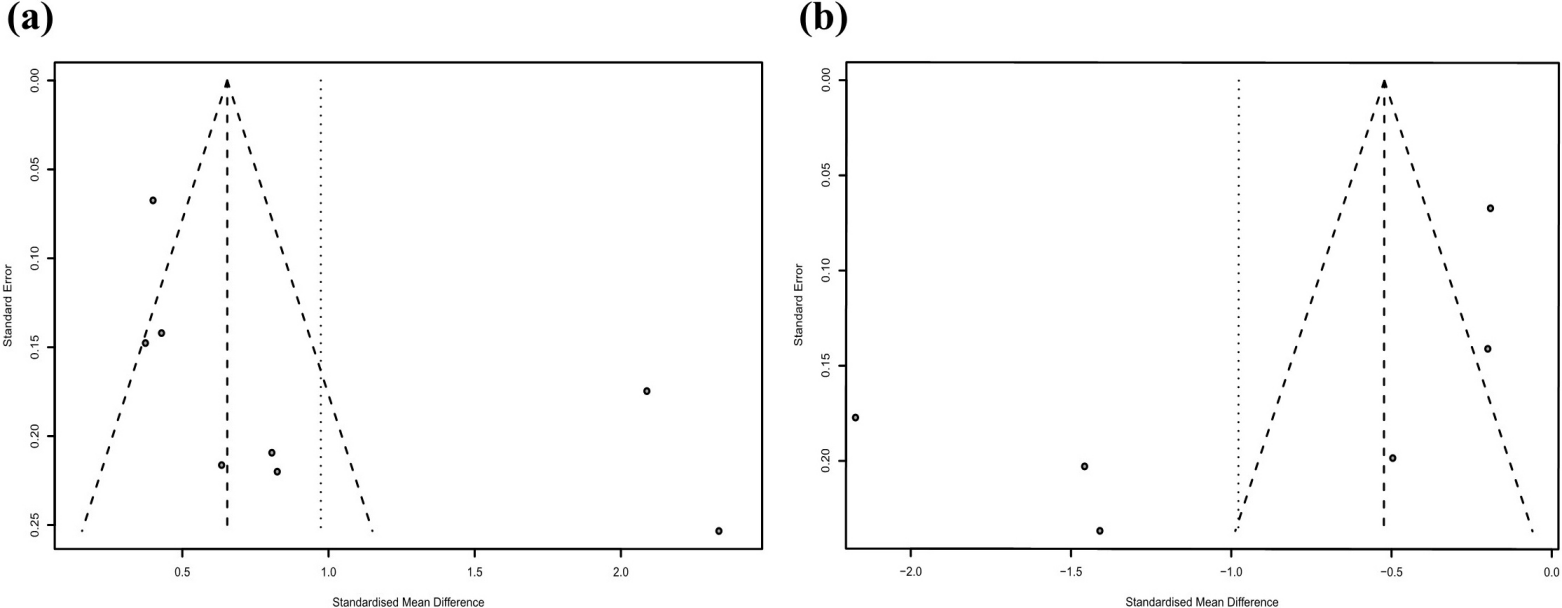
**Funnel plots: (a) knowledge; (b) decision conflict**.

**Fig. 6.**
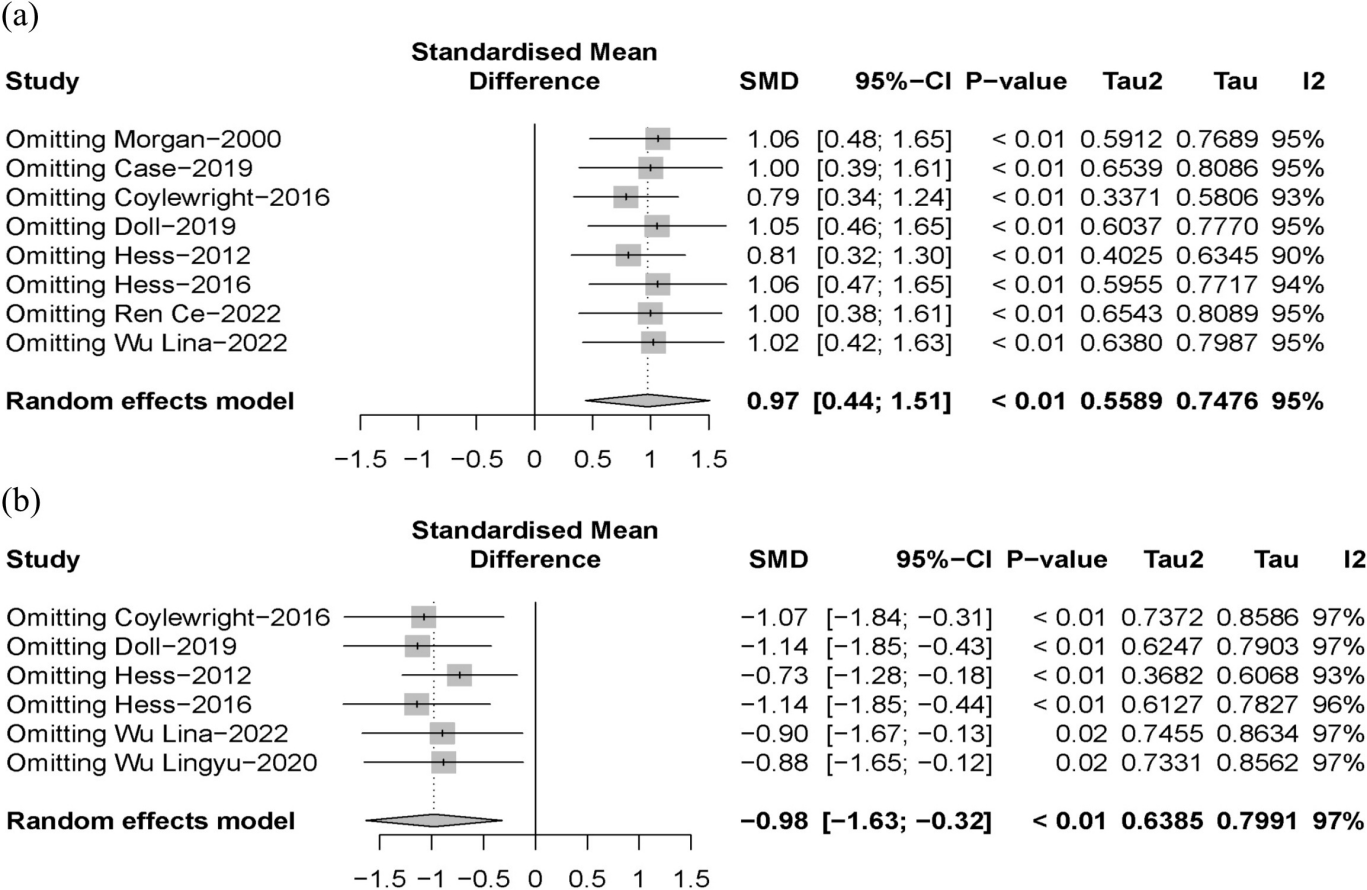
**Sensitivity analysis: (a) knowledge; (b) decision conflict**. SMD, standardized mean difference.

The funnel plots were slightly asymmetric (Fig. 5), but the Egger’s test suggested that there was no evidence for potential publication bias regarding knowledge (*t* = 1.90; *p* = 0.1058) and decision conflict (*t* = –2.15; *p* = 0.0983). The results showed that there was a high heterogeneity in the knowledge, decision conflict, satisfaction, patient involvement, and trust. Therefore, to ensure the stability of our results, we conducted a sensitivity analysis by omitting each study in turn. The results did not change even after removing one or two studies, although heterogeneity was reduced to varying degrees (Fig. 6).

## 4. Discussion

We reviewed the role of SDM interventions in the form of PtDAs in CHD patients’ medical decision-making, including 2133 participants from 10 studies. Overall, the quality of the included studies was good. The research involved various forms of PtDAs, including manuals, videos, etc. The main content included information and treatment plans for CHD patients. Five studies were conducted in the United States, and six were published after 2019, exhibiting typical spatiotemporal differences that may be related to factors such as healthcare policies and cultural differences. After all, the USA government promoted the development of SDM in the medical field by making policies and providing funds [31]. It is necessary to conduct additional multicenter, large-sample studies to explore the underlying reasons for differences in the development of shared decision-making.

SDM has been highlighted as a desirable approach to clinical counseling [37]. However, the definition of SDM in CHD needs to be clarified. In particular, the role and function of patients and their families in decision-making have yet to be clearly defined when patients lose decision-making ability and have limited decision-making time. The 10 included studies mainly focused on CHD treatment choice, symptom management, and low-risk screening without involving the evaluation of SDM in emergency CHD situations. Most importantly, no studies show whether patients, their families, and surgeons are willing to use SDM in the complex surgical environment of CHD. Even in critical situations, medical staff should try to inform the risks and benefits of treatment. By reviewing the application of SDM in low-risk chest pain, AF, and syncope, Probst MA [38] concluded that using SDM in specific and appropriate acute cardiovascular disease scenarios is both feasible and morally meaningful. Kunneman M *et al*. [39] reduced the time for informed consent for stroke prevention in patients with AF by 1.1 minutes overall through SDM intervention based on electronic information technology. Undoubtedly, this brief minute is vital for some CHD patients. SDM based on preference is especially meaningful, in situations such as choosing between surgery and death, selecting a surgical option selection under emergency conditions, and determining whether the surgical effect is acceptable based on individual patient differences. SDM is crucial for patient diagnosis, treatment, and medical quality [40].

The PtDA is an effective intervention tool that conveys complex information to patients in an easy-to-understand way and is used to facilitate the SDM process. Subgroup analysis showed that PtDA has no significant difference in knowledge improvement among different countries, indicating that PtDA is universal and effective under different cultural and policy backgrounds. The primary analysis found that SDM intervention in the form of PtDAs improved patients’ knowledge. In this study, the content of decision support tools includes basic knowledge of coronary heart disease, risk and benefits comparison of alternative options. Providing sufficient and easy-to-understand information can reduce patient information acquisition biases and provide a foundation for analyzing decision-making content. Communication is the premise for all medical decisions [41]. The Three-talk Model of SDM and the Interprofessional SDM model have been used to optimize the medical decision processes for limb rehabilitation [42] and intensive care unit patient care [43], respectively, and both have achieved good results. To ensure patient safety, it is necessary to use effective communication methods to optimize the SDM process and improve patients’ understanding of disease knowledge.

In addition, SDM alleviates decision-making conflicts in CHD patients. Analyzing the pros and cons of alternative solutions is the most concerning issue for patients. It is also one of the critical factors that cause uncertainty in patient decision-making [36]. The PtDAs included in the study presented patients’ medical information using visualizations such as tables and drawings. SDM optimizes the medical decision-making process by determining the decision-making situation, sharing knowledge, expressing preferences, making decisions, providing clear guidance to patients, and effectively reducing decision-making conflicts. However, some studies suggest that SDM cannot solve decision conflicts [25]. Lack of information, unclear values, and pressure are the leading causes of patient decision-making conflicts [25]. Decision aids can only provide patients with relevant information and help them clarify their values, but their effectiveness is limited. When patients face external pressure, decision-making conflicts still exist. In the implementation process of SDM, the support of doctors can help patients better participate in decision-making. On the other hand, society needs to establish a social security system to reduce the impact of economic and work conditions on decision-making.

For secondary outcomes, we found that SDM increased participation in CHD patients. “Patient/family member participation” has always been the core concept of SDM, which improves decision-makers’ understanding of patients’ conditions, provides more opportunities to communicate with doctors, and increases the affection between doctors and patients. SDM is carried out with the help of doctors, who are likely to be an essential factor affecting the quality of decision-making. As a tool to promote decision-making practice, PtDAs cannot replace the work of doctors but play a more auxiliary role [16]. We should be wary of over-reliance on PtDAs. Emphasizing the critical role of doctors’ participation in decision-making and incorporating their training provides a reference for the further development of SDM.

SDM has improved the satisfaction of CHD patients, which contradicts research results such as He *et al*. [44], and the reason may be related to the type of disease. Compared to the long recovery period of breast morphology after breast reconstruction surgery, the implementation of surgery can quickly alleviate discomfort symptoms such as chest tightness and pain in patients with CHD. Logical, objective, and fair PtDAs enable decision-makers to make high-quality decisions, and patients are more satisfied with the whole decision-making process. Trust is the foundation of the doctor-patient relationship. We did not find that SDM significantly affected doctor-patient trust, which may be disappointing. Trust generation is comprehensively influenced by the external environment, personal character, and habits [42]. It is difficult to significantly improve patients’ trust in doctors only through short-term communication. Also, we cannot deny that the poor implementation of SDM may have compromised trust improvement by participating clinicians. SDM may bring minor enhancements in communication but also stimulate more significant doubts about the authority of clinicians [45].

Medical decision-making is an iterative process that changes as a patient’s disease progresses. The premise of SDM is that patients are aware that they need to make decisions together with their doctors and are prepared [15]. We found that a few studies [28, 29, 30, 33] reported that patients were willing to accept the intervention of SDM. We only described it qualitatively and did not include it in the meta-analysis. These patients are primarily stable CHD with sufficient decision-making time and ability. However, saving lives is essential for patients with CHD who need to be rescued, and a doctor-led decision-making approach is more appropriate [15]. Overall, decision-making time, ability, condition, and understanding of the disease may affect patients’ views on SDM.

It should be noted that anxiety is the main reason for the increase in cardiovascular risk in CHD patients. Although two studies have shown that SDM can improve emotions, this conclusion must be approached cautiously. Negative emotions are a long-term process influenced by personal experience, social support, and partner attitudes [46]. PtDAs are designed to provide treatment-related information to improve the quality of decision-making, not specifically for mental health. Considering the impact of emotions on the onset of CHD and postoperative rehabilitation, it is necessary to establish a comprehensive psychological intervention plan to improve patients’ negative emotions. Similarly, although four studies reported that patients in the intervention group had a higher quality of life after surgery, the improvement of postoperative somatic symptoms is related to the efficacy of surgery.

SDM interventions conducted in PtDAs format may positively impact decision quality and treatment outcomes for CHD patients. From the systematic evaluation of the included research content in this study, some specific clinical issues still warrant further investigation. Firstly, in terms of disease characteristics, the development trajectory of CHD is uncertain, and patients may experience comorbidities and repeated admissions [2]. Considering that CHD may affect the function of heart valves, the use of PtDAs to assist patients in exploring disease prioritization or surgical strategies when facing two different types of surgical treatment simultaneously is a topic that requires in-depth research. CHD patients who receive SDM interventions during their initial visit may have a certain understanding of disease knowledge and treatment methods. When patients are re-admitted, the content of SDM may be more inclined towards the effectiveness of previous treatment plans, whether further adjustments or replacements are needed, rather than repeating disease knowledge. The SDM on the diagnosis and treatment of CHD has been applied in clinical practice [27, 28, 29, 30, 31, 32, 33, 34, 35, 36], and it is necessary to further investigate the different application backgrounds of CHD, develop PtDAs for specific occasions, and expand the application scope of SDM. Considering the low health literacy of middle-aged and elderly CHD patients [36], regular health education activities should be held, and visual PtDAs such as concise manuals and videos should be developed to improve compliance. Different cultural backgrounds and religious beliefs should also be considered. For example, under the influence of the “family group concept” in China, when patients and their families have disagreements, they should be further assisted in reaching a consensus and making satisfactory treatment choices. Finally, it is necessary to build a SDM culture. We need to further ensure the development of SDM in CHD through pipeline strategies such as strengthening doctor training, building doctor-patient communication channels, optimizing decision-making processes, paying attention to patient feedback, and timely improvement.

To our knowledge, this was the first evidence to summarize the effectiveness of SDM interventions in the form of PtDAs in patients with CHD in the world. This study systematically reviewed and analyzed the results related to PtDAs to provide suggestions for future development. Some limitations should be acknowledged in this study. First, a high heterogeneity was observed in this study. The heterogeneity might result from the differences in the form, content, and measures scales of PtDAs, national health policies, culture context, and the degree of patient participation in decision-making. Thus, the results of this meta-analysis should be interpreted cautiously. Second, there may be some potential sources of bias in the study, including selection bias and publication bias. However, we ensured that no potential biases could affect the validity of the meta-analysis results by sensitivity analyses, creating funnel plots, and performing the Egger’s test. Third, a few studies did not report the development process of PtDAs, and we were unable to know whether it had been verified and met the International Patient Decision Aid Standards (IPDAS), which may limit the impact of the study protocol on patients with CHD. Finally, although sensitive methods are used to search the literature, including only English and Chinese may result in omitting some literature.

## 5. Conclusions

SDM interventions may be a promising clinical practice in the area of decision-making in CHD patients. However, the results should be interpreted with caution due to the variability of PtDAs and SDM content. In addition, although SDM has been proven to be applicable to emergency heart disease, none of the included studies is to evaluate the application of SDM in CHD emergencies. Further evaluation of the effectiveness of SDM intervention in the form of PtDAs in different environments in patients with CHD is needed.

## Abbreviations

AF, atrial fibrillation; CABG, coronary artery bypass grafting; CHD, coronary heart disease; CVD, cardiovascular disease; DAs, decision aids; ED, emergency department; HF, heart failure; MDs, mean differences; OMT, optimal medical therapy; PCI, percutaneous coronary intervention; PtDAs, Patient decision aids; RCTs, randomized controlled trials; SDM, shared decision-making; SMDs, standardized mean differences.

## Supplementary Material

Supplementary material associated with this article can be found, in the online version, at https://doi.org/10.31083/j.rcm2408246.
