# Supplementary material for: Interventions to Facilitate Shared Decision-Making Using Decision Aids with Coronary Heart Disease Patients: Systematic Review and Meta-Analysis
Source: Rev Cardiovasc Med. 2023 Aug 25;24(8):246. doi: 10.31083/j.rcm2408246 (PMC11266786; doi:10.31083/j.rcm2408246)
Supplement: Supplementary file 1 [file 2153-8174-24-8-246-s1.zip › 2153-8174-24-8-246-s1/Supplementary Material 2.docx]

**Search terms**

PubMed

("Myocardial Ischemia"[MeSH Terms] OR "coronary Disease"[Title/Abstract] OR "angina pectoris"[Title/Abstract] OR "acute coronary syndrome"[Title/Abstract] OR "myocardial infarction"[Title/Abstract] OR "coronary heart disease*"[Title/Abstract] OR "coronary artery disease*"[Title/Abstract]) AND ("Decision Trees"[MeSH Terms] OR "Decision Support Techniques"[MeSH Terms] OR "decision support systems, clinical"[MeSH Terms] OR "Decision Making"[MeSH Terms] OR "Audiovisual Aids"[MeSH Terms] OR ("decision aid*"[Title/Abstract] OR "decision support*"[Title/Abstract] OR "decision tool*"[Title/Abstract] OR "decision instrument*"[Title/Abstract] OR "decision technolog*"[Title/Abstract] OR "decision technique*"[Title/Abstract] OR "decision system*"[Title/Abstract] OR "decision program*"[Title/Abstract] OR "decision algorithm*"[Title/Abstract] OR "decision method*"[Title/Abstract] OR "decision intervention*"[Title/Abstract] OR "decision process*"[Title/Abstract] OR "decision material*"[Title/Abstract] OR "risk communication tool*"[Title/Abstract] OR "risk assessment tool*"[Title/Abstract] OR "risk information tool*"[Title/Abstract] OR "patient decision*"[Title/Abstract] OR "informed decision*"[Title/Abstract] OR "informed choice*"[Title/Abstract] OR "shared decision making"[Title/Abstract] OR "risk communication method*"[Title/Abstract] OR "risk assessment method*"[Title/Abstract])) AND ("randomized controlled trial"[Publication Type] OR "controlled clinical trial"[Publication Type] OR ("randomized"[Title/Abstract] OR "trial"[Title/Abstract] OR "placebo"[Title/Abstract] OR "randomly"[Title/Abstract]))

Web of Science

#1: ((((((TS=("Myocardial Ischemia")) OR TS=("coronary Disease")) OR TS=("angina pectoris")) OR TS=("acute coronary syndrome")) OR TS=("myocardial infarction")) OR TS=("coronary heart disease*")) OR TS=("coronary artery disease*")

#2: TS=( Decision Tree* OR Decision Support Technique* OR Decision Support Systems, Clinical OR Audiovisual Aid* OR decision* aid* OR decision* support* OR decision* tool* OR decision* instrument* OR decision* technolog* OR decision* technique* OR decision* system* OR decision* program* OR decision* algorithm* OR decision* process* OR decision*method* OR decision* intervention* OR decision* material* OR informed choice* OR shared decision making)

#3: TS=(random* controlled trial OR controlled clinical trial OR random* OR trial OR placebo )

#4: #3 AND #2 AND #1

Embase

('myocardial ischemia'/exp OR 'coronary disease'/exp OR 'angina pectoris'/exp OR 'acute coronary syndrome'/exp OR 'myocardial infarction'/exp OR 'coronary heart disease*' OR 'coronary artery disease*') AND ('decision trees'/exp OR 'decision support techniques'/exp OR 'decision support systems, clinical'/exp OR 'decision making'/exp OR 'audiovisual aids'/exp OR 'decision aid*' OR 'decision support*' OR 'decision tool*' OR 'decision instrument*' OR 'decision technolog*' OR 'decision technique*' OR 'decision system*' OR 'decision program*' OR 'decision algorithm*' OR 'decision method*' OR 'decision intervention*' OR 'decision process*' OR 'decision material*' OR 'risk communication tool*' OR 'risk assessment tool*' OR 'risk information tool*' OR 'patient decision*' OR 'informed decision*' OR 'informed choice*' OR 'shared decision making'/exp OR 'risk communication method*' OR 'risk assessment method*') AND ('randomized controlled trial'/exp OR 'controlled clinical trial'/exp OR 'randomized' OR 'trial'/exp OR 'placebo'/exp OR 'randomly')

Cochrane

#1: MeSH descriptor: [Myocardial Ischemia] explode all trees

#2: ("coronary disease"):ti,ab,kw OR ("coronary heart disease*"):ti,ab,kw OR ("coronary artery disease*"):ti,ab,kw OR ("chest pain"):ti,ab,kw

#3: MeSH descriptor: [Decision Support Techniques] this term only

#4: MeSH descriptor: [Decision Support Systems, Clinical] this term only

#5: MeSH descriptor: [Decision Trees] this term only

#6: MeSH descriptor: [Decision Making] this term only

#7: MeSH descriptor: [Audiovisual Aids] this term only

#8: ("decision aid*" OR "decision support*" OR "decision tool*" OR "decision instrument*" OR "decision technolog*" OR "decision technique*" OR "decision system*" OR "decision program*" OR "decision algorithm*" OR "decision method*" OR "decision intervention*" OR "decision process*" OR "decision material*" OR "risk communication tool*" OR "risk assessment tool*" OR "risk information tool*" OR "patient decision*" OR "informed decision*" OR "informed choice*" OR "shared decision making" OR "risk communication method*" OR "risk assessment method*"):ti,ab,kw

#9: #1 OR #2

#10: #3 OR #4 OR #5 OR #6 OR #7 OR #8

#11: #9 AND #10
